# Intra-Articular Collagen Injections for Osteoarthritis: A Narrative Review

**DOI:** 10.3390/ijerph20054390

**Published:** 2023-03-01

**Authors:** Domiziano Tarantino, Rosita Mottola, Stefano Palermi, Felice Sirico, Bruno Corrado, Rossana Gnasso

**Affiliations:** Department of Public Health, University of Naples Federico II, 80131 Naples, Italy

**Keywords:** collagen, collagen injections, intra-articular injections, infiltrative treatment, osteoarthritis, knee osteoarthritis

## Abstract

Osteoarthritis (OA) is the most frequent degenerative progressive joint disease worldwide, with the hand, hip, and knee being the most-affected joints. Actually, no treatment can alter the course of OA, and therapy is directed at reducing pain and improving function. The exogenous administration of collagen has been investigated as a possible symptomatic adjuvant or stand-alone treatment for OA. The aim of this review is to assess if intra-articular collagen administration can be considered as a valid and safe therapeutic option for OA. A search in the main scientific electronic databases to identify the available scientific articles about the effects of intra-articular collagen as an OA treatment was performed. The results of the seven included studies showed that the intra-articular administration of collagen may stimulate chondrocytes to produce hyaline cartilage and hinder the normal inflammatory response leading to fibrous tissue formation, reducing symptoms, and improving functionality. The use of type-I collagen as an intra-articular treatment for knee OA was found not only to be effective, but also safe with negligible side effects. The reported findings are strongly promising, highlighting the need for further high-quality research to confirm the consistency of these findings.

## 1. Introduction

Osteoarthritis (OA) is the most frequent degenerative progressive joint disease worldwide, concerning middle-aged and elderly individuals, with a higher incidence in women. OA mainly affects both small and large diarthrodial joints with the hand, hip, and knee being the most-affected ones [1]. Economic and social costs of OA are high, and its impact on patients’ quality of life (QoL) may be devastating in terms of limitation of the activities of daily living (ADL) and absence from work [2,3,4].

Different aetiologies lead to the development of OA, but there are similar biological and clinical factors that cause a reduction in the articular range of motion (ROM), muscle weakness, pain, and that increase disability worsening the QoL [5]. Radiologically, OA is characterized by the incremental erosion of the articular cartilage, especially of its outermost portion, with consequent subchondral sclerosis and cyst formation, joint space narrowing, synovial inflammation, and marginal osteophyte development [6].

Currently, no treatment can alter the course of OA, and therapy is directed at reducing pain and improving function [7,8]. Medications including nonsteroidal anti-inflammatory drugs (NSAIDs), acetaminophen, duloxetine, opioids, topical NSAIDs, and capsaicin are effective in reducing symptoms [9]. Intra-articular injections are usually carried out using corticosteroid [10], hyaluronic acid (HA) [11,12,13,14,15], ozone [16], plasma rich in growth factor [17], and platelet-rich plasma [18,19].

During the onset of OA, an acute inflammatory process is established that tends to become chronic: chondrocytes are exposed to the joint cavity, osmotic pressure in the articular cartilage changes, and proteoglycans migrate progressively into the joint space, hindering the natural healing stages [20,21]. Pro-inflammatory factors such as TNF-β, IL-6, IL-1α, and IL-1β are released, stimulating cartilage-degrading enzymes such as a disintegrin and metalloproteinase with thrombospondin motifs (ADAMTS), and matrix metalloproteinases (MMPs) [22]. These enzymes cause the degradation of the extracellular matrix (ECM), including collagen [23]. For this reason, the exogenous administration of collagen has been investigated as a possible symptomatic adjuvant or stand-alone treatment for OA [24,25].

Collagen is a major structural protein in the human body, representing the main component of connective tissue and constituting approximately 45–75% of dry weight in the ligaments, tendons, and cartilage [26]. Collagen fibres are a major component of the extracellular matrix that supports most tissues and plays an important role in cell structure [26].

The in vitro exposure of animal or human synovial and cartilage cells to collagen preparations, with different degrees of hydrolysation or polymerization, was shown to induce the up-regulation of chondrocyte proliferation and production of cartilage extracellular matrix proteins, to increase the production of hyaluronic acid, and to reduce the release of inflammatory mediators [27,28,29,30,31]. For example, purified porcine atelocollagen, a soluble type-I collagen with good biocompatibility, minor immunogenicity, a long half-life, and high resistance to enzymatic degradation, was shown to directly reduce inflammation and to promote tissue repair, acting as a scaffold for regeneration reaction around the damaged areas [32,33,34].

In rabbit models with induced femoral groove defects, the intra-articular administration of atelocollagen was shown to be effective; Suh et al. showed a restored cartilage layer at weeks 3 and 12 after the injections and, histologically, a regenerated cartilage like that of the normal articular cavity [26].

Naraoka et al. [35] found that periodical injections of collagen tripeptide and collagen tripeptide plus hyaluronic acid delayed the progression of cartilage degeneration in early OA induced by anterior cruciate ligament transection in rabbits.

Given these promising in vitro and in vivo findings about the effects of collagen on OA models and the paucity of knowledge about its use in humans for treating OA, the aim of this review is to assess whether intra-articular collagen injections can be considered as a valid and safe therapeutic option for OA. To best of our knowledge, this is the first study that reviews the use of intra-articular collagen injections for OA in humans.

## 2. Materials and Methods

All the procedures related to this review were organized and reported after performing a search in the main scientific electronic databases (PubMed, Scopus, etc.) to identify the articles about the effects of intra-articular collagen as an OA treatment, with no restrictions of time and language.

For the purposes of our review, we used several combinations of the following keywords: collagen, injection, intra-articular treatment, infiltrative treatment, osteoarthritis. Editorials, technical notes, letters to authors, narrative reviews, systematic review articles, and articles that did not report any clinical outcomes were excluded. Furthermore, articles involving surgical approaches, physiotherapy, physical therapies, and other kind of therapies were excluded.

Only articles that assessed the outcomes of intra-articular collagen injections in humans (alone or in combination with other types of drugs) for OA were selected.

## 3. Results

The results of the research lead to the selection of seven articles; all the articles found evaluated the effects of intra-articular collagen injections for knee OA. A summary of the outcomes of the selected studies is reported in Table 1.

In 2009, Furuzawa-Carballeda et al. [27] conducted a prospective, randomized, double-blind placebo-controlled in vivo clinical trial on the effectiveness and safety of type-I polymerized collagen in patients affected by knee OA. Primary and secondary outcomes such as the Lequesne Index (LI) (i.e., an index of severity of knee OA, with higher scores indicating worse severity), Western Ontario and McMaster University Osteoarthritis (WOMAC) Index (i.e., an index that quantifies knee pain during the ADL, with higher scores indicating worse knee pain and impairment while performing the ADL), and pain intensity on a visual analogue scale (VAS) (higher scores indicate worse pain) were evaluated. Enzyme immunoassays were employed to quantify urinary levels of C-terminal crosslinking telopeptide of type-II collagen (CTXII) and serum high-sensitivity C-reactive protein (hsCRP). These tests were used to monitor the progression of the joint damage.

Type-I polymerized collagen injections emerged as being well tolerated and effective, since patients showed a statistically significant clinical improvement at six months compared with baseline and placebo in almost all primary and secondary endpoints. The beneficial effects of type-I polymerized collagen injections lasted throughout the entire follow-up. Furthermore, in the placebo group, a threefold rise in CTXII was found, indicating the progression of joint damage and cartilage degradation. Moreover, patients who received polymerized collagen decreased the use of NSAIDs at statistically significant levels compared with baseline and placebo group.

These promising findings were confirmed by Furuzawa-Carballeda et al. [36] in 2012 in a double-blind and placebo-controlled trial on patients affected by symptomatic knee OA who underwent arthroscopic lavage followed by type-I polymerized collagen injections.

Patients treated with collagen were compared with baseline and placebo at the 3-month follow-up, showing clinical benefits and elevated response rates for primary and secondary outcomes. Patients also reported VAS improvement and IL-1β, and IL-10 peripheral-expressing cell reduction. A noteworthy result was the decreased level of urinary CTX-II of 44% in patients who received collagen injections, demonstrating an improvement in the joint damage. Moreover, patients treated with collagen considerably reduced NSAID consumption at statistically significant levels. These outcomes proved that type-I polymerized collagen administration is able to reduce the response time to the pharmacological treatment after arthroscopic surgery, favouring the decrease in proteolytic enzymes, synovial reaction, and pro-inflammatory factors, optimizing the articular ROM [36].

In another double-blind randomized active-controlled clinical trial performed in 2016 by Martin Martin et al. [37], the intra-articular administration of type-I hydrolysed porcine collagen was compared to the intra-articular HA administration in patients affected by knee OA. At the three- and six-month follow-ups, collagen injections showed the same beneficial effects and safety as intra-articular HA, whose use is widespread for treating OA [37].

The authors finally stated that the reduced cost of collagen compared to low- or high-MW HA could allow a greater use of intra-articular collagen as an alternative therapeutic option to HA for OA, resulting in an NSAID intake reduction, as well as social cost reduction due to working days lost and caregivers’ time off work.

In 2019, Lee and colleagues [38] conducted a multicentre, randomized, double-blind study on the intra-articular administration of type-I atelocollagen in subjects affected not only by OA, but also by chondromalacia and other cartilage defects, with patients suffering from knee OA representing the most consistent group (86%). Their outcomes showed that the resulting VAS scores of the collagen group were not different to those of the placebo group at 4 and 12 weeks, but a statistically significant improvement in the collagen group was reported at 24 weeks. The WOMAC and Short Form Health Survey 36 (SF-36) (i.e., a patient-reported survey of patient health, with the higher the score, the less the disability) scores also showed significant improvement after the intra-articular injection of atelocollagen, although scores between the collagen group and placebo were similar; both WOMAC and SF-36 were not significantly different between the two groups at 4, 12, and 24 weeks. Moreover, 72 subjects in the collagen group were satisfied with the clinical benefit, while only 57 patients from the placebo group showed satisfaction. Equally, practitioners were satisfied with 73 better outcomes from patients in the collagen group and only 61 from the placebo group. Their results demonstrated that intra-articular atelocollagen administration is able to reduce knee pain not only for OA, but also for other pathologic cartilage conditions such as chondromalacia [38].

Borja-Flores et al. [39], in a cohort study published in 2020, evaluated the efficacy of the intra-articular administration of type-I polymerized collagen in preventing and/or delaying total knee replacement surgery (TKR) among subjects affected by symptomatic mild-to-moderate knee OA. At a 60-month follow-up, 95.5% of the subjects who underwent type-I polymerized collagen injection showed positive outcomes and none needed to resort to TKR due to the persistence of pain and knee stiffness. Five subjects (1.6%) resorted to arthroscopy, seven (2.3%) subjects to high tibial osteotomy (HTO), and two subjects (0.6%) to anterior cruciate ligament (ACL) reconstruction. Instrumental investigations supported and confirmed the clinical benefits of collagen injections: radiographic images of 42 knees were obtained at baseline and at 60 months, and were evaluated for Kellgren Lawrence (KL) classification, showing a remarkable difference between baseline and follow-up with the preservation of the joint space. Patients who underwent intra-articular polymerized-collagen injections reported a statistically significant improvement in the primary outcomes, optimizing the OA course, reducing pain and functional disability. At 60 months after the therapeutic effects, a 98.8% freedom from surgical procedures such as arthroscopy and TKR was reported. Interestingly, the same percentage of freedom from surgery was highlighted in both obese and normal-weighted people.

In 2019, De Luca and colleagues [40] conducted a functional in vitro investigation and a pilot retrospective clinical study on the effectiveness of intra-articular hydrolysed collagen to treat knee OA. In the in vitro part of the study, they took human chondrocytes from five donors affected by KL grade IV OA who underwent total hip arthroplasty with the aim of assessing the growth and viability of the chondrocytes exposed to hydrolysed collagen. The expression of MMP1/MMP3 and TIMP1/TIMP3 (their inhibitors) was evaluated using RT-PCR, while the expression of trophic factors such as IGF-I and TGFβ1 and angiogenic factors such as VEGF was evaluated via ELISA assays. After 28 days of hydrolysed collagen exposition, the extracellular matrix (ECM) constitution was studied through the Bern score, while the collagen deposition was studied using immunostaining. The hydrolysed collagen did not induce any change in the release of the aforementioned factors, but it positively affected the structure of chondrocytes, presenting smaller cell density, higher morphology, and better ECM deposition.

In the pilot study, subjects were enlisted to receive three injections of an innovative formulation of collagen characterized by hydrolysed collagen of bovine (and not porcine like the previously reported studies) origin with an MW < 3 kiloDalton (kDa).

Both VAS and LI scores decreased significantly after the first injection, but not after the second injection, and remained stable at the 6-month follow-up. WOMAC scores significantly decreased after the second injection compared to those at baseline, and after the third injection, they were lower than after the second one. All the scores collected were not significantly modified at the 6-month follow-up from those collected after the third injection. In conclusion, the authors stated that this formulation of hydrolysed collagen may stimulate chondrocytes to produce hyaline cartilage and to reduce fibrous tissue formation, and it was found to be effective and safe in the treatment of mild–moderate knee OA [40].

These positive findings of this study were later confirmed by Volpi et al. [1] in a multicentric retrospective clinical study in 2020, showing that the same formulation of hydrolysed collagen (bovine origin with an MW < 3 kDa) and the same injection protocol (three injections) is well-tolerated without any significant adverse effects, also reporting stronger evidence on a larger sample of patients than previously published by De Luca et al. [40].

At the last follow-up, 70 subjects reported a 50% decrease in the LI, a 50% decrease in the VAS score at rest and moving, and a ≥50% reduction in WOMAC sub-scores and total score. Similar to what was stated by De Luca et al. [40], the authors concluded that the intra-articular administration of hydrolysed collagen is a safe and effective short-term adjuvant in the treatment of symptomatic knee OA [1].

## 4. Discussion

OA frequently affects the knee joint, with remarkable social costs and a strong impact on patients’ QoL, resulting in reduced capacity to perform the ADL. Different therapeutic strategies have been tested so far, and all of them were effective in the short term. Therefore, adjuvant non-pharmacological therapies may be tempting since they allow the combination of different specific benefits with different durations.

The results of the present narrative review showed that the intra-articular administration of collagen may stimulate chondrocytes to produce hyaline cartilage and hinder the normal inflammatory response, leading to fibrous tissue formation, reducing symptoms, and improving functionality. The use of type-I collagen as an intra-articular treatment for knee OA was found not only to be effective, but also safe with negligible side effects. Furthermore, the intra-articular administration of type-I collagen was shown to increase the benefits of arthroscopic lavage when administered after it, as well as being able to significantly delay the need for joint replacement such as TKR.

The studies by De Luca et al. and Volpi et al. [1,40] were the only ones concerning hydrolysed collagen of bovine origin and low MW (<3 kDa), and the effects associated with its intra-articular use for knee OA. The study conducted by Volpi et al. [1] demonstrated, on a larger sample of patients, the effectiveness of hydrolysed collagen and the safety of treating knee OA in patients presenting a BMI < 30 and a grade 1 to 4 of KL. These findings confirmed those of De Luca et al. [40]. However, some differences emerged between the outcomes of the two studies. When low-MW hydrolysed collagen was injected on a large-scale study, it resulted in a lower decrease in VAS at rest, with only a 50% reduction (half of the 100% showed by De Luca and colleagues). Moreover, a minor reduction compared to the outcomes reported by De Luca et al. [40] was also observed in the WOMAC stiffness subscale (50% Volpi vs. 75% De Luca). For all the other scores evaluated, the differences between the two studies were neglectable. Nevertheless, the lower percentage values found by Volpi et al. [1] did not reflect a lesser clinical benefit, which remained considerable in most patients. Moreover, compared to all the other studies discussed, the studies conducted by De Luca et al. [40] and Volpi et al. [1] were the only ones that provided a three-dose infiltrative protocol, and not five or more weekly doses, with this different frequency in the collagen administration not resulting in any less clinical benefit.

Further comparing the outcomes between the included studies, the outcomes regarding VAS, LI, and WOMAC scores demonstrated a similar improvement between the studies of De Luca et al. and Volpi et al. [1,40] and the studies by Furuzawa-Carballeda et al. [27,36] in all the three indexes, with the LI and WOMAC scores of the first two studies [1,40] being better than the last two at six months [27,36].

A similar improvement was reported in the studies by De Luca et al. and Volpi et al. [1,40] and Martin Martin et al. [37] concerning VAS and LI scores, even if the LI scores collected in the first two studies [1,40] were better than in the last one at six months [37].

Again, a similar improvement regarding the global assessment of disease activity change at the end of the treatment measured using the Likert score (LS) (the higher the score, the better the change in disease activity) was found in the studies by Furuzawa-Carballeda [27,36] and the one by Borja-Flores [39]. While in the first two studies, the patient LS was already significant at three and two months (respectively), in the last study, it became statistically significant only at 12 months. Regarding the physician LS, in the first two studies, it became statistically significant at four and three months (respectively), while in the last study, it became statistically significant at six months.

It is not clear whether these differences were affected by the different frequency of administration of collagen even in the studies from the same authors (12 vs. 6 injections in studies by Furuzawa-Carballeda), or the different composition of the collagen injected. Indeed, the collagen administrated by Furuzawa-Carballeda [27,36] was a γ-irradiated mixture of atelopeptidic porcine type-I collagen and polyvinylpyrrolidone, the one used by Martin Martin [37] was a pure type-I collagen with a much heavier (300 kDa) MW, and the hydrolysed formulation of collagen used by De Luca et al. and Volpi et al. [1,40] was a low-molecular weight (<3 kDa) type-I collagen.

Furthermore, apart from the number of injections administrated and the type of collagen used, the results of the selected studies confirmed that all types of collagen formulation can alleviate painful symptoms of knee OA, improving QoL and reducing physical limitations, thanks to the regenerative and proliferative stimulus given by collagen to the chondrocytes [27,36]. Furthermore, as reported by Borja-Flores et al. [39], polymerized type-I collagen is the cheapest cost–benefit alternative compared to NSAIDs for the symptomatic treatment of knee OA.

The beneficial effects of type-I polymerized collagen were also found in laboratory and radiologic data.

Furuzawa-Carballeda et al. [36] found that at 48 weeks after the last injection, a three-fold rise in CTX-II was found only in the placebo group. The authors explained that the CTX-II decrease found in patients treated with intra-articular polymerized collagen was associated with a lower pro-inflammatory cytokine (IL-1β- and TNF-α-) amount with an increase in CD4 T-regulatory cells and IL-10-producing cells [27,36].

Finally, concerning the radiological evaluation, Borja-Flores et al. [39] showed positive results at a 60-month follow-up, with most of the subjects treated with intra-articular type-I polymerized collagen advancing from stage II to III, but not from III to IV in the KL grading scale. Furthermore, the use of intra-articular type-I polymerized collagen prevented remarkable changes in the knee joint width in the medial, lateral, and patellofemoral compartments, suggesting no radiological aggravation.

The main limitation of this review is the very small number of articles found in the scientific literature about the effects and safety of intra-articular type-I collagen injections for OA in humans. The current poor knowledge about its therapeutic potential on OA could limit its diffusion as an intra-articular treatment, leading physicians to prefer drugs with a stronger scientific rationale, such as HA.

Furthermore, the infiltrative protocols and the type of collagen used differed from each other, so it is not possible to draw definitive conclusions about which protocol and which kind of collagen provides the best benefits.

## 5. Conclusions

The results of the present review showed that the intra-articular administration of collagen may represent a valid therapeutic option for knee OA. The use of type-I collagen as an intra-articular treatment for knee OA was found not only to be effective, but also safe with negligible side effects.

Given the promising results of the articles included in this review, more in vitro and in vivo high-quality research (such as randomized clinical trials) should be performed for a further understanding of the beneficial effects of type-I collagen as an intra-articular treatment for OA, since there is still not enough knowledge about its effectiveness.

Furthermore, all the selected studies regarded the use of intra-articular type-I collagen only for knee OA, so it would be interesting to extend its use to other joints commonly affected by OA.

## Figures and Tables

**Table 1:** A summary of the outcomes of the selected studies.

Study and Authors	Collagen Used	KL Grade	Groups	Intervention	Timing of Clinical Assessment	Outcomes	Adverse Effects
Furuzawa-Carballeda et al. [27]	Type-I polymerized collagen	N/D	Collagen (*n* = 27) vs. placebo (*n* = 26)	12 injections (weeks 1, 2, 3, 4, 6, 8, 10, 12, 14, 16, 20 and 24)	Baseline, 6 months, 12 months	↓ LI ↓ WOMAC ↓ VAS ↓ NSAIDs use ↑ LS	Injection site pain lasting <24 h 2/27 (collagen group) cases of aseptic acute arthritis
Furuzawa-Carballeda et al. [36]	Type-I polymerized collagen	III-IV	Collagen (*n* = 10) vs. placebo (*n* = 9) after arthroscopic lavage	6 injections (1 after arthroscopic lavage, then 1 a week for 5 weeks 6 weeks after surgery)	Baseline, 3 months, 6 months	↓ LI ↓ WOMAC ↓ VAS ↓ NSAIDs use ↑ LS ↑ evaluation of drug efficacy	Injection site pain lasting <24 h
Martin Martin et al. [37]	Type-I hydrolysed collagen	II-III	Collagen (*n* = 32) vs. HA (*n* = 32)	5 injections (1 a week for 5 weeks in a row)	Baseline, 3 months, 6 months	↓ LI ↓ VAS ↓ Pain killer consumption ↑ SF-36 questionnaire	1/32 (collagen group) moderate post-injection reaction
Lee et al. [38]	Type-I atelocollagen	I-II-III	Collagen (*n* = 101) vs. placebo (*n* = 99)	1 injection	Baseline, 1 month, 3 months, 6 months	↓ VAS ↓ WOMAC ↑ SF-36 questionnaire	11/101, of which 55% knee pain
Borja-Flores et al. [39]	Type-I polymerized collagen	II-III	Collagen (*n* = 309)	6 injections (1 a week for 6 weeks in a row)	Baseline, 6–11 months, 12–35 months, 36–48 months, 49–60 months	↓ VAS ↓ WOMAC ↑ functional disability ↑ time of surgical referral of TKA	Injection site pain <24 h in all patients
De Luca et al. [40]	Type-1 hydrolysed collagen	I-II-III-IV	Collagen (*n* = 20)	3 injections (weeks 1, 15, 45)	Baseline, 15 days, 1 month, 6 months	↓ LI ↓ WOMAC ↓ VAS	None
Volpi et al. [1]	Type-1 hydrolysed collagen	I-II-III-IV	Collagen (*n* = 70)	3 injections (weeks 1, 15, 45)	Baseline, 15 days, 1 month, 6 months	↓ LI ↓ WOMAC ↓ VAS	None

KL = Kellgren Lawrence; LI = Lequesne Index; WOMAC = Western Ontario and McMaster Universities Osteoarthritis Index; VAS = Visual Analogue Scale; NSAIDs = Non-Steroidal Anti-Inflammatory Drugs; LS = Likert Score; SF-36 = Short Form Health Survey 36; TKA = Total Knee Arthroplasty.

## Data Availability

Not applicable.

## References

[1] Volpi P., Zini R., Erschbaumer F., Beggio M., Busilacchi A., Carimati G. (2021). Effectiveness of a Novel Hydrolyzed Collagen Formulation in Treating Patients with Symptomatic Knee Osteoarthritis: A Multicentric Retrospective Clinical Study. Int. Orthop..

[2] Xie F., Kovic B., Jin X., He X., Wang M., Silvestre C. (2016). Economic and Humanistic Burden of Osteoarthritis: A Systematic Review of Large Sample Studies. Pharmacoeconomics.

[3] Cross M., Smith E., Hoy D., Nolte S., Ackerman I., Fransen M., Bridgett L., Williams S., Guillemin F., Hill C.L. (2014). The Global Burden of Hip and Knee Osteoarthritis: Estimates from the Global Burden of Disease 2010 Study. Ann. Rheum. Dis..

[4] Fayaz A., Croft P., Langford R.M., Donaldson L.J., Jones G.T. (2016). Prevalence of Chronic Pain in the UK: A Systematic Review and Meta-Analysis of Population Studies. BMJ Open.

[5] Felson D.T., Lawrence R.C., Dieppe P.A., Hirsch R., Helmick C.G., Jordan J.M., Kington R.S., Lane N.E., Nevitt M.C., Zhang Y. (2000). Osteoarthritis: New Insights. Part 1: The Disease and Its Risk Factors. Ann. Intern. Med..

[6] Buckwalter J.A., Mankin H.J. (1998). Articular Cartilage: Degeneration and Osteoarthritis, Repair, Regeneration, and Transplantation. Instr. Course Lect..

[7] Bannuru R.R., Vaysbrot E.E., Sullivan M.C., McAlindon T.E. (2014). Relative Efficacy of Hyaluronic Acid in Comparison with NSAIDs for Knee Osteoarthritis: A Systematic Review and Meta-Analysis. Semin. Arthritis Rheum..

[8] Altman R.D., Bedi A., Karlsson J., Sancheti P., Schemitsch E. (2016). Product Differences in Intra-Articular Hyaluronic Acids for Osteoarthritis of the Knee. Am. J. Sport. Med..

[9] McAlindon T.E., Bannuru R.R., Sullivan M.C., Arden N.K., Berenbaum F., Bierma-Zeinstra S.M., Hawker G.A., Henrotin Y., Hunter D.J., Kawaguchi H. (2014). OARSI Guidelines for the Non-Surgical Management of Knee Osteoarthritis. Osteoarthr. Cartil..

[10] da Costa B.R., Hari R., Jüni P. (2016). Intra-Articular Corticosteroids for Osteoarthritis of the Knee. JAMA.

[11] He W.-W., Kuang M.-J., Zhao J., Sun L., Lu B., Wang Y., Ma J.-X., Ma X.-L. (2017). Efficacy and Safety of Intraarticular Hyaluronic Acid and Corticosteroid for Knee Osteoarthritis: A Meta-Analysis. Int. J. Surg. Lond. Engl..

[12] Wang C.-T., Lin J., Chang C.-J., Lin Y.-T., Hou S.-M. (2004). Therapeutic Effects of Hyaluronic Acid on Osteoarthritis of the Knee. A Meta-Analysis of Randomized Controlled Trials. J. Bone Jt. Surg. Am..

[13] Pellegrino R., Di Iorio A., Brindisino F., Paolucci T., Moretti A., Iolascon G. (2022). Effectiveness of Combined Extracorporeal Shock-Wave Therapy and Hyaluronic Acid Injections for Patients with Shoulder Pain Due to Rotator Cuff Tendinopathy: A Person-Centered Approach with a Focus on Gender Differences to Treatment Response. BMC Musculoskelet. Disord..

[14] Pellegrino R., Paolucci T., Brindisino F., Mondardini P., Di Iorio A., Moretti A., Iolascon G. (2022). Effectiveness of High-Intensity Laser Therapy Plus Ultrasound-Guided Peritendinous Hyaluronic Acid Compared to Therapeutic Exercise for Patients with Lateral Elbow Tendinopathy. J. Clin. Med..

[15] Pellegrino R., Brindisino F., Barassi G., Sparvieri E., DI Iorio A., de Sire A., Ruosi C. (2022). Combined Ultrasound Guided Peritendinous Hyaluronic Acid (500-730 Kda) Injection with Extracorporeal Shock Waves Therapy vs. Extracorporeal Shock Waves Therapy-Only in the Treatment of Shoulder Pain Due to Rotator Cuff Tendinopathy. A Randomized Clinical Trial. J. Sport. Med. Phys. Fit..

[16] Raeissadat S.A., Tabibian E., Rayegani S.M., Rahimi-Dehgolan S., Babaei-Ghazani A. (2018). An Investigation into the Efficacy of Intra-Articular Ozone (O2-O3) Injection in Patients with Knee Osteoarthritis: A Systematic Review and Meta-Analysis. J. Pain Res..

[17] Sánchez M., Fiz N., Azofra J., Usabiaga J., Aduriz Recalde E., Garcia Gutierrez A., Albillos J., Gárate R., Aguirre J.J., Padilla S. (2012). A Randomized Clinical Trial Evaluating Plasma Rich in Growth Factors (PRGF-Endoret) versus Hyaluronic Acid in the Short-Term Treatment of Symptomatic Knee Osteoarthritis. Arthroscopy.

[18] Ayhan E., Kesmezacar H., Akgun I. (2014). Intraarticular Injections (Corticosteroid, Hyaluronic Acid, Platelet Rich Plasma) for the Knee Osteoarthritis. World J. Orthop..

[19] de Sire A., Lippi L., Mezian K., Calafiore D., Pellegrino R., Mascaro G., Cisari C., Invernizzi M. (2022). Ultrasound-Guided Platelet-Rich-Plasma Injections for Reducing Sacroiliac Joint Pain: A Paradigmatic Case Report and Literature Review. J. Back Musculoskelet. Rehabil..

[20] Maldonado M., Nam J. (2013). The Role of Changes in Extracellular Matrix of Cartilage in the Presence of Inflammation on the Pathology of Osteoarthritis. BioMed Res. Int..

[21] Wu J.P., Kirk T.B., Zheng M.H. (2008). Study of the Collagen Structure in the Superficial Zone and Physiological State of Articular Cartilage Using a 3D Confocal Imaging Technique. J. Orthop. Surg..

[22] Kapoor M., Martel-Pelletier J., Lajeunesse D., Pelletier J.-P., Fahmi H. (2011). Role of Proinflammatory Cytokines in the Pathophysiology of Osteoarthritis. Nat. Rev. Rheumatol..

[23] Mobasheri A., Bay-Jensen A.-C., van Spil W.E., Larkin J., Levesque M.C. (2017). Osteoarthritis Year in Review 2016: Biomarkers (Biochemical Markers). Osteoarthr. Cartil..

[24] Abe T., Yamada H., Nakajima H., Kikuchi T., Takaishi H., Tadakuma T., Fujikawa K., Toyama Y. (2003). Repair of Full-Thickness Cartilage Defects Using Liposomal Transforming Growth Factor-Beta1. J. Orthop. Sci. Off. J. Jpn. Orthop. Assoc..

[25] Hurtig M., Chubinskaya S., Dickey J., Rueger D. (2009). BMP-7 Protects against Progression of Cartilage Degeneration after Impact Injury. J. Orthop. Res. Off. Publ. Orthop. Res. Soc..

[26] Suh D.S., Yoo J.C., Woo S.H., Kwak A.S. (2021). Intra-Articular Atelocollagen Injection for the Treatment of Articular Cartilage Defects in Rabbit Model. Tissue Eng. Regen. Med..

[27] Ohara H., Iida H., Ito K., Takeuchi Y., Nomura Y. (2010). Effects of Pro-Hyp, a Collagen Hydrolysate-Derived Peptide, on Hyaluronic Acid Synthesis Using in Vitro Cultured Synovium Cells and Oral Ingestion of Collagen Hydrolysates in a Guinea Pig Model of Osteoarthritis. Biosci. Biotechnol. Biochem..

[28] Furuzawa-Carballeda J., Muñoz-Chablé O.A., Macías-Hernández S.I., Agualimpia-Janning A. (2009). Effect of Polymerized-Type I Collagen in Knee Osteoarthritis. II. In Vivo Study. Eur. J. Clin. Investig..

[29] Comblain F., Dubuc J.-E., Lambert C., Sanchez C., Lesponne I., Serisier S., Henrotin Y. (2016). Identification of Targets of a New Nutritional Mixture for Osteoarthritis Management Composed by Curcuminoids Extract, Hydrolyzed Collagen and Green Tea Extract. PLoS ONE.

[30] Furuzawa-Carballeda J., Muñoz-Chablé O.A., Barrios-Payán J., Hernández-Pando R. (2009). Effect of Polymerized-Type I Collagen in Knee Osteoarthritis. I. In Vitro Study. Eur. J. Clin. Investig..

[31] Oesser S., Seifert J. (2003). Stimulation of Type II Collagen Biosynthesis and Secretion in Bovine Chondrocytes Cultured with Degraded Collagen. Cell Tissue Res..

[32] Hanai K., Kojima T., Ota M., Onodera J., Sawada N. (2012). Effects of Atelocollagen Formulation Containing Oligonucleotide on Endothelial Permeability. J. Drug Deliv..

[33] Chang S.-W., Flynn B.P., Ruberti J.W., Buehler M.J. (2012). Molecular Mechanism of Force Induced Stabilization of Collagen against Enzymatic Breakdown. Biomaterials.

[34] Seong H., Kim R.K., Shin Y., Lee H.W., Koh J.C. (2020). Application of Purified Porcine Collagen in Patients with Chronic Refractory Musculoskeletal Pain. Korean J. Pain.

[35] Naraoka T., Ishibashi Y., Tsuda E., Yamamoto Y., Kusumi T., Toh S. (2013). Periodic Knee Injections of Collagen Tripeptide Delay Cartilage Degeneration in Rabbit Experimental Osteoarthritis. Arthritis Res. Ther..

[36] Furuzawa-Carballeda J., Lima G., Llorente L., Nuñez-Álvarez C., Ruiz-Ordaz B.H., Echevarría-Zuno S., Hernández-Cuevas V. (2012). Polymerized-Type I Collagen Downregulates Inflammation and Improves Clinical Outcomes in Patients with Symptomatic Knee Osteoarthritis Following Arthroscopic Lavage: A Randomized, Double-Blind, and Placebo-Controlled Clinical Trial. Sci. World J..

[37] Martin Martin L.S., Massafra U., Bizzi E., Migliore A. (2016). A Double Blind Randomized Active-Controlled Clinical Trial on the Intra-Articular Use of Md-Knee versus Sodium Hyaluronate in Patients with Knee Osteoarthritis (“Joint”). BMC Musculoskelet. Disord..

[38] Lee H.S., Oh K.J., Moon Y.W., In Y., Lee H.J., Kwon S.Y. (2021). Intra-Articular Injection of Type I Atelocollagen to Alleviate Knee Pain: A Double-Blind, Randomized Controlled Trial. Cartilage.

[39] Borja-Flores A., Macías-Hernández S.I., Hernández-Molina G., Perez-Ortiz A., Reyes-Martínez E., Belzazar-Castillo de la Torre J., Ávila-Jiménez L., Vázquez-Bello M.C., León-Mazón M.A., Furuzawa-Carballeda J. (2020). Long-Term Effectiveness of Polymerized-Type I Collagen Intra-Articular Injections in Patients with Symptomatic Knee Osteoarthritis: Clinical and Radiographic Evaluation in a Cohort Study. Adv. Orthop..

[40] De Luca P., Colombini A., Carimati G., Beggio M., de Girolamo L., Volpi P. (2019). Intra-Articular Injection of Hydrolyzed Collagen to Treat Symptoms of Knee Osteoarthritis. A Functional In Vitro Investigation and a Pilot Retrospective Clinical Study. J. Clin. Med..

